# Heparin, Heparan Sulphate and the TGF-β Cytokine Superfamily

**DOI:** 10.3390/molecules22050713

**Published:** 2017-04-29

**Authors:** Chris C. Rider, Barbara Mulloy

**Affiliations:** Centre for Biomedical Sciences, School of Biological Sciences, Royal Holloway University of London, Egham, Surrey TW20 0EX, UK; b.mulloy@imperial.ac.uk

**Keywords:** heparin, heparan sulphate, TGF-β, bone morphogenetic protein (BMP), growth and differentiation factor (GDF), GDNF, BMP antagonists, noggin, sclerostin, gremlin

## Abstract

Of the circa 40 cytokines of the TGF-β superfamily, around a third are currently known to bind to heparin and heparan sulphate. This includes TGF-β1, TGF-β2, certain bone morphogenetic proteins (BMPs) and growth and differentiation factors (GDFs), as well as GDNF and two of its close homologues. Experimental studies of their heparin/HS binding sites reveal a diversity of locations around the shared cystine-knot protein fold. The activities of the TGF-β cytokines in controlling proliferation, differentiation and survival in a range of cell types are in part regulated by a number of specific, secreted BMP antagonist proteins. These vary in structure but seven belong to the CAN or DAN family, which shares the TGF-β type cystine-knot domain. Other antagonists are more distant members of the TGF-β superfamily. It is emerging that the majority, but not all, of the antagonists are also heparin binding proteins. Any future exploitation of the TGF-β cytokines in the therapy of chronic diseases will need to fully consider their interactions with glycosaminoglycans and the implications of this in terms of their bioavailability and biological activity.

## 1. The TGF-β Cytokine Superfamily

The vertebrate TGF-β superfamily comprises some 40 cytokines, which regulate cellular activities encompassing proliferation, differentiation, and survival in diverse cell types (for reviews see [1,2,3]). They are thus important in tissue morphogenesis and development, as well as in regulating tissue homeostasis in the adult. Accordingly some members of the superfamily have come to the fore in the field of regenerative medicine, such as in the maintenance and subsequent differentiation of embryonic stem cells [1]. Moreover, aberrant signalling by TGF-β family cytokines occurs in a range of chronic diseases including tissue fibrosis and various cancers, and thus an understanding of their roles and modes of action is of relevance in effort to develop effective therapies for such diseases.

Within the superfamily, there are three TGF-βs [2], four activins [3], four neurotropic factors [4] and 21 bone morphogenetic proteins (BMPs)/growth and differentiation factors (GDFs) [5]. Across the superfamily a number of different receptors and signalling pathways are employed, but the canonical signalling pathway is via a complex of Type I and Type II serine/threonine kinases, cell surface receptors which activate cytoplasmic Smad transcription factors. The field of TGF-β family cytokine signalling and receptor usage has recently been reviewed elsewhere [6].

**Figure 1:**
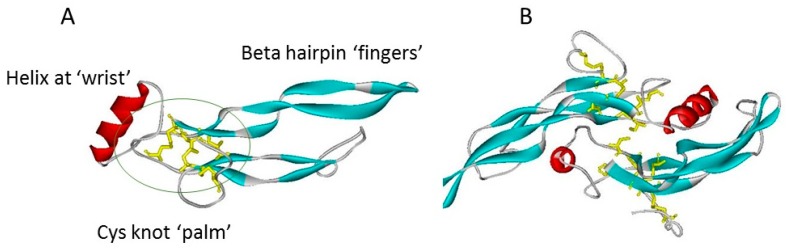
The TGF-β superfamily cystine knot fold, as typified by TGF-β1 (co-ordinates from 1KLC.pdb). Protein chains are shown in ribbon format: β-strands are blue, helices red, turns green; cystines are shown in yellow stick format. (**A**) TGF-β1 monomer showing the “hand” structures, with the cystine knot indicated by a green ellipse. (**B**) TGF-β1 dimer, the “wrist” of each hand is cupped in the other subunit. The view of the dimer is rotated by 90° with respect to the plane of 1A. The interchain disulphide bridge is visible in the centre of the structure.

A further superfamily feature is a commonality of structure based on a cystine knot motif (Figure 1) Although variations occur, this typically contains within a sequence of some 110 residues, seven cysteines, of which six form intrachain disulphide bridges with the connectivity of CyS1-CyS5, CyS2-Cys6, and Cys3-CyS7. These covalent bridges give rise to a true knot structure, as CyS -2, -3, -6 and -7 together with neighbouring amino acids in the polypeptide chain, form an eight-membered circle of covalently linked residues, through which the CyS1-CyS5 linkage passes [7]. This covalent knot holds together a polypeptide fold in which two narrow β-strand finger loops project in near parallel from one face of a short α-helix [8,9]. A simply analogy of this structure is that of a hand (Figure 1A) bearing only two slightly curved fingers (the β-strand loops). The cystine knot region provides the palm, with the α-helix comprising the heel or wrist of the hand. Many but not all of the superfamily members exist as covalently linked dimers, held together by an interchain disulphide bridge between the two Cys4 residues which are un-partnered within the monomeric structure. This covalent bridge gives rise to an elongated, offset, face-to-face dimer in which the paired β-strand finger loops of each subunit extend away from the each other at opposite ends of the structure (Figure 1B).

## 2. Protein Antagonists of TGF-β Cytokines

The activities of a number of the TGF-β superfamily members are tightly regulated, in part through several secreted antagonist proteins which bind certain of these cytokines with high affinity, inhibiting cell surface receptor engagement, and thereby signalling. These antagonists include Noggin, Twisted Gastrulation (TSG), Chordin and Chordin-like proteins, and Follistatin (FST) and Follistatin like proteins (FSTLs). These antagonist proteins were initially recognised through their important roles in embryonic development [10]. A further family of seven antagonists is the Cerberus or Dan (CAN) family, which comprises Cerberus, Coco, Dan, Gremlin (also referred to as Gremlin-1), PRDC/gremlin-2, Sclerostin and USAG-1. Remarkably, the CAN family antagonists are also TGF-β superfamily members, by virtue of possessing the characteristic eight-membered cystine-knot domain [7]. The non-CAN antagonists, TSG, Chordin, and Noggin, but not Follistatin, possess more diverse variants of the cystine-knot motif within their larger structures, and therefore are distant members of the TGF-β superfamily [11].

## 3. Interactions of TGF-β Cytokines with Heparin and Heparan Sulphate

**Figure 2:**
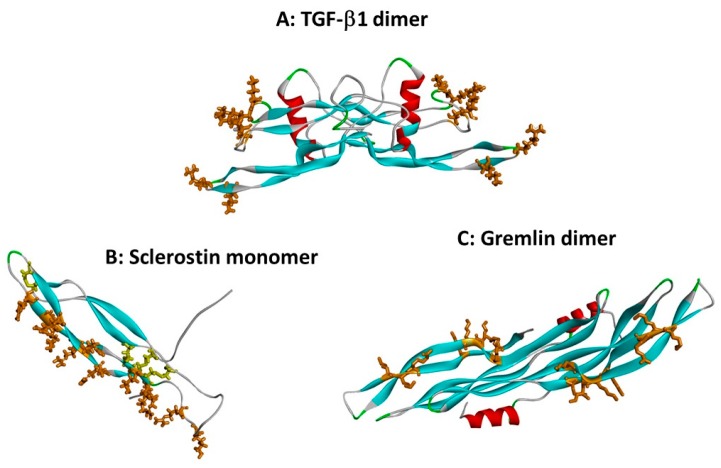
Heparin binding sites on TGF-β superfamily cytokines. Protein chains are shown as in Figure 1, and heparin binding site basic residues are shown in brown stick format. (**A**) The dimer of TGF-β1 (co-ordinates from 1KLC.pdb). Residues K25, R26, K31, K37, R94 and R97 form a discontinuous heparin binding site at the tips of the “fingers” [13]. (**B**) Sclerostin monomer (one of the NMR ensemble in 2K8P.pdb) with heparin-binding residues in brown. Cystine residues are shown in yellow stick format. Loop 3 (the second β-strand loop) and Loop 2 are both involved; residues K99, R102, R114, R116, R119, R131, R133, K134, R136, K142, K144 and R145 form a linear heparin binding site capable of accommodating a heparin dodecamer [26]. (**C**) The dimer of the CAN BMP antagonist gremlin (co-ordinates from 5AEJ.pdb). Here, the heparin binding residues are located largely along the second “finger”, as for sclerostin; in the dimer both copies of the heparin binding site are on the concave face. Residues K145, K147, K148, K167, K168, K169, K174 and R177 have been identified as forming the heparin binding site [20]. The mode of dimerisation and the location of the heparin binding site both differ from those of TGF-β1.

It has emerged that a number of the TGF-β superfamily cytokines bind to the sulphated polysaccharide heparin and heparan sulphate (HS) with interactions strong enough to be relevant at physiological ionic strength and pH. This was first established in the case of TGF-β1 by McCaffrey et al. [12]. Subsequently Lyon et al. [13] showed that heparin and highly sulphated liver HS bind both human TGF-β1 and TGF-β2, but not TGF-β3. On this basis, they proposed a discontinuous heparin/HS binding site based on basic residues located at the tips of the first finger loops (Figure 2A). The heparin binding affinity data for these two TGF-βs, and other superfamily members, are presented in Table 1.

**Figure 3:**
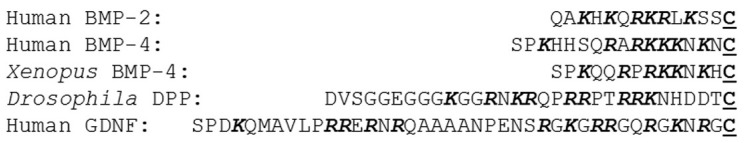
*N*-terminal sequences of some heparin binding BMPs. The sequences are shown ending with the first cysteine (shown in bold underlined font at the right hand side) of the knot domain. The basic residues lysine and arginine are highlighted in bold italics, and sequence regions experimentally implicated in heparin binding are boxed.

The 21 TGF-β superfamily cytokines that are designated BMPs and GDFs can be grouped into subfamilies of between two and four members on the basis of sequence homology, and thereby presumably recent evolutionary divergence [5]. BMP-2 and -4 comprise one such subfamily. They possess amino terminal sequences, 13 and 15 residues in length, respectively, upstream of the first cysteine of their knot domains which are rich in the basic amino acids, Arg and Lys. In both cases, as previously reviewed [27], these contain heparin binding sites (see Figure 3). Mammalian BMP-2 and -4 show surprisingly high homology to the *Drosophila* morphogen decapentaplegic (Dpp). Dpp similarly binds to the cell surface HS proteoglycans Dally and dally-like, which are the *Drosophila* orthologs of mammalian glypicans [28].

BMPs and GDFs of other subfamilies also bind to heparin and HS. Two highly homologous BMPs, BMP-6 [29] and BMP-7 [15,30,31], have both been shown to bind to heparin. The binding sites within these two BMPs are yet to be established, but their aminoterminal sequences are quite distinctive from those of BMP-2 and -4, being nearly three times longer. GDF-9 (BMP-15) [17] and most recently GDF-5 (BMP-14) [16], each representing a further subfamily, have also been shown to bind to heparin and HS. In neither case has the binding site been established. Predictive molecular docking calculations have indicated that a combination of unstructured *N*-terminal sequences and residues in the Cys knot region may combine to form heparin binding sites [32].

The four TGF-β superfamily neurotrophic factors are glial cell line-derived neurotrophic factor (GDNF), artemin, persephin and neurturin. All four regulate neuronal differentiation, share high sequence homology and signal at least in part through the cell surface tyrosine kinase Ret [33]. GDNF, artemin and neurturin have all been shown to bind to heparin [18] and to the HS proteoglycan syndecan-3 [19]. In the case of GDNF, the heparin binding site has been mapped to a 16-residue sequence containing 7 Arg and Lys residues immediately *N*-terminal to the first cysteine of the knot domain [18]. Although the *N*-terminal sequences of the other neurotrophins are considerably shorter than that of GDNF, those of artemin and neurturin, but not persephin are also enriched in basic residues. A notable feature of the binding of GDNF to heparin is its particular dependence on the presence of 2-*O*-sulphates [34].

## 4. Interactions of BMP Antagonist Proteins with Heparin and HS

Amongst the CAN family of antagonists, gremlin-1, gremlin-2, PRDC and sclerostin all bind to heparin and HS [20,21,22,26,35]. In each case the respective heparin binding sites lie within the cystine knot domain and involve exposed basic amino acid sidechains within the second β-strand finger loop [20,22,26,35]. For Dan and Cerberus, using molecular docking simulations we predicted a lack of affinity for heparin (see supplementary data, Rider and Mulloy, 2010 [5]), and for DAN this has since been confirmed experimentally [22]. We are unaware of any reports on the heparin/HS binding of Coco although on secretion it is retained on cell surfaces [36], a behaviour consistent with interaction with HS proteoglycans.

**Figure 4:**
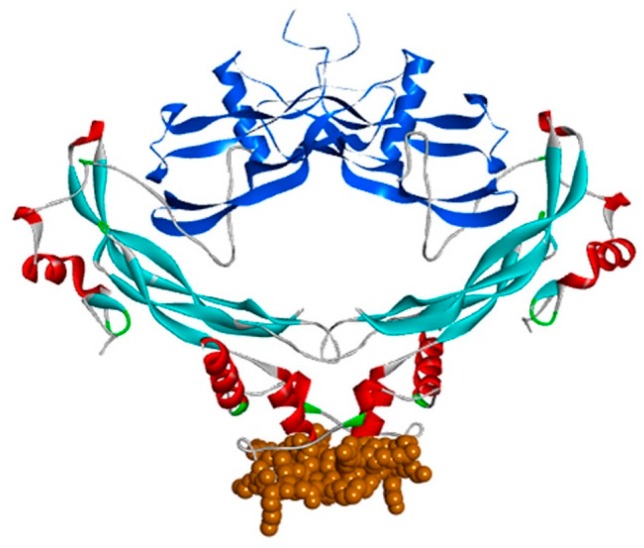
The heparin binding site of noggin in the noggin-BMP-7 complex (co-ordinates from 1M4U.pdb). Noggin is shown as described in Figure 1, with amino acids 133–144, encompassing a cluster of eight basic arginine and lysine residues, shown in brown CPK format; BMP-7 is shown in blue ribbon format.

**Figure 5:**
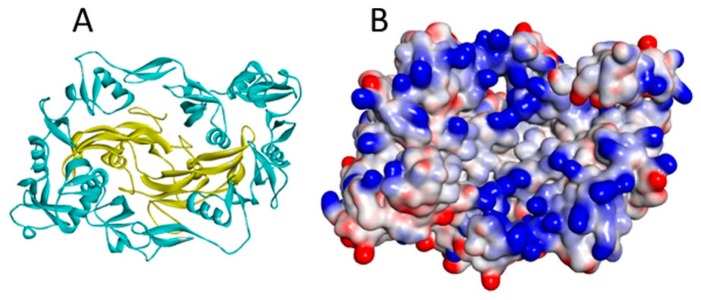
(**A**) The myostatin (yellow ribbon)/follistatin (turquoise ribbon) complex (3HH2.pdb); and (**B**) the same complex shown as a surface coloured according to interpolated charge (positive is blue, negative is red). Though myostatin does not have a heparin binding site, basic residues on its surface are located close to the follistatin binding site in the complex, increasing total affinity for heparin [40].

The more distantly related, non-CAN BMP antagonists are also heparin/HS binding proteins. This is well established for Noggin, which has a heparin binding site rich in basic residues aminoterminal to the cystine-knot domain [23] and lying within the “wrist” region of the polypeptide fold (Figure 4). Follistatin has been well characterised as a heparin binding protein that binds to cell surfaces through interaction with HS [37]. The heparin binding site has been identified as the sequence rich in basic amino acids between Lys75 and Arg6 [38]. The finding that follistatin complexed with myostatin (GDF-8) has much higher affinity for heparin than either of the two individual protein components [25] may be explained by the juxtaposition in the complex of the positively charged faces of myostatin and follistatin to form a single enhanced heparin binding site [39] (Figure 5). The follistatin–activin A complex also has enhanced affinity for heparin as measured by surface plasmon resonance, but is less stable to increased ionic strength than the follistatin-myostatin complex [25]. On the other hand, the follistatin related protein, follistatin-like 3, has no heparin binding site, and does not acquire heparin-binding properties when complexed with myostatin [40]. Chordin too binds heparin and HS, but the particular binding site involved has yet to be elucidated [24]. Thus, taken overall, current data reveal that the interaction with HS proteoglycans seems to be an important characteristic of the majority of the various BMP antagonist proteins. Moreover, at least in some instances, the complexes formed by BMPs and antagonists appear to have increased affinity for heparin, compared to the uncomplexed proteins.

## 5. Heparin/HS Binding Sites of the TGF-β Cytokines

Returning to the more typical TGF-β superfamily members possessing the eight-residue circle cystine-knot, according to current knowledge, of the circa 40 mammalian proteins, two TGF-βs, six BMPs/GDFs, three out of four neurotrophins, and three of the CAN antagonists are known to bind to heparin/HS, around one third of the total number of proteins. With further study, this proportion is likely to rise, although given the paucity of clustered basic residues in some of the protein sequences, heparin/HS binding would appear to be far from a universal behaviour within this superfamily. Interestingly, within these proteins that do bind, their heparin/HS binding sites show considerable diversity. Thus, BMP-2, BMP-4, and GDNF have binding sites immediately aminoterminal to the cystine knot domain (Figure 3), whereas TGF-β1 and -β2 utilise the tips of their first β-strand finger loops (Figure 2A), and the CAN antagonists bind primarily via the surfaces of their second β-strand finger loops (Figure 2B,C). This leads to the conjecture that heparin/HS binding is a property acquired by certain members following the considerable divergence of this superfamily which occurred with the evolutionary emergence of the vertebrates. It would therefore appear to represent a fine-tuning mechanism for the biological activities of such proteins. It will be interesting to see whether future studies are able to determine whether or not each of the differing binding site locates can be associated with a different functional outcome of heparin/HS binding.

The Norrie disease protein, norrin, is an outlier of the cystine-knot family, possessing three β-strand loops per monomeric subunit. It activates the canonical Wnt/β-catenin pathway by binding to the the Frizzled4 cell surface receptor and the co-receptor low-density lipoprotein receptor related protein 5/6 (Lrp5/6). Like Wnt, it also interacts with cell surface HS proteoglycans. Mutational and structural studies of norrin have highlighted arginines 107, 109 and 115, which are located at the tip of the third β-strand loop as key residues in the GAG-binding site [41]. The triple substitution of these arginines with non-basic residues abolishes signalling activity and, interestingly, such a mutation occurs naturally, and is one causative of Norrie disease. These observations strongly implicate cell surface HS binding as critical for the normal functioning of norrin.

## 6. Effect of Heparin/HS Binding on TGF-β Cytokine Activity

Given that the TGF-β superfamily cytokines are relatively small, soluble glycoproteins, binding to HS proteoglycans of the extracellular matrix or on cell surfaces will have a major effect of restricting their diffusion away from sites of secretion within the tissues. This is well established for Dpp, the *Drosophila* homologue of BMP-2 and -4. Within the developing wing, a high concentration of Dpp defines the anterior-posterior axis, and a large body of work has established that the glypican HS proteoglycans dally and dally-like are not only responsible for maintaining this morphogenic gradient, but for transporting dpp across fields of cells to generate this gradient (reviewed by Nybakken and Perrimon [42]). Since there are close vertebrate homologues of all of the macromolecules involved in this mechanism it is reasonable to expect that BMP morphogenetic gradients are established and maintained by glypicans in higher organisms too.

Murine GDNF is another TGF-β cytokine for which there is clear evidence of the importance of HS in maintaining high localised concentrations of morphogens. In the initial stages of kidney formation, GDNF is expressed in the embryonic metanephric blastema and serves as a chemoattractant for cells of the ureteric bud which express the GDNF receptors. Contact between cells of these two embryonic structures results in the cellular condensation and proliferation events which lead to kidney formation [43]. The key role of GDNF in these events is revealed by the *GDNF^−/−^* mouse, in which there is a total absence of kidneys. Strikingly, this phenotype is recapitulated by homozygous knock-out of the gene encoding HS 2-*O*-sulphotransferase [44]. Since the binding of GDNF to heparin shows an unusually high dependence on the presence of 2-*O*-sulphate groups [34], these various studies support the paradigm that in the wild-type embryonic mouse, 2-*O*-sulphate replete HS is responsible for maintaining secreted GDNF within the metanephric blastema at concentrations sufficient to activate signalling in the arriving ureteric bud cells. In the absence of 2-*O*-sulphated HS, inadequate GDNF would be retained in this microenvironment. Further support for the role of HS proteoglycans in restricting the diffusion of GDNF within the tissues arises from studies of the administration of the recombinant cytokine into rat brain, whereupon a mutant lacking the heparin/HS binding domain was seen to diffuse more freely than the wild type cytokine [45].

Beyond the effect of HS binding restricting the diffusion of these cytokines, there is the issue of how binding to HS might affect their biological activities. Potentially the binding of small cytokine to bulky glycosaminoglycan chains might obscure their receptor binding sites, thereby inhibiting signalling activity. Alternatively, as is well established within the fibroblast growth factor (FGF) cytokine family, heparin and HS might serve as co-receptors, promoting signalling [46,47]. For FGF1, an initial step by which heparin promotes signalling activity is the formation of cytokine dimers through polypeptide-polysaccharide interactions. This dimerisation then facilitates receptor engagement [48]. Heparin-induced dimerisation is also a mechanism for promoting FGF2 signalling [49]. Since most TGF-β cytokines exist as disulphide-bridged dimers in circulation, a dimerisation function for GAG appears unnecessary with this superfamily. Moreover, as the locations of heparin/HS binding sites vary from one cytokine to another within the TGF-β family (see Figure 2), it may not necessarily be the case that GAG binding will affect TGF-β cytokine activity in a single, uniform way.

In one of the earliest studies of the effects of heparin/HS binding on BMP activity, a mutant of BMP-2 with abrogated heparin binding was found to be more active than the wild-type cytokine in chick limb bud assays [14]. This indicates that heparin binding is not obligatory for receptor activation, and thus that the co-receptor role for heparin/HS observed with FGFs does not apply for BMP-2. However the activity of wild type BMP-2 was increased by the addition of exogenous heparin [14]. Thus, interaction with extracellular matrix HS would appear to modulate the bioavailability of BMP-2. Essentially similar outcomes have been observed in subsequent studies [31,50,51,52,53]. Similar modulation of BMP-4 activity by heparin and HS has also been shown [54]. Of these studies, Jiao et al., have considered that the retention of a BMP on HS close to cell surfaces will not only retain the cytokine near its receptors facilitating signalling, but also in the vicinity of cellular internalisation mechanisms, promoting internalisation and turnover [50]. Thus, HS binding may facilitate both BMP signalling and turnover.

In embryonic stem cell differentiation exogenous heparin and highly sulphated HS have been shown to restore BMP-4 signalling to enable haematopoietic differentiation in HS-deficient cells [55]. Although this may appear to be consistent with a possible co-receptor function, a further study ascribed this effect to HS stabilising BMP-4 against degradation [56]. This indicates that the influence of HS on BMP signalling may well be dependent on cellular context. With BMP-7, biological activity in inhibiting cell proliferation in the subventricular zone of adult brain, a major site of neurogenesis, is also dependent on the presence of cell surface HS [57].

Beyond the BMPs, removal of heparin/HS binding for GDNF has no apparent effect on its receptor binding and cellular activity [18]. However the in vivo neuroprotective activity of GDNF in a rat model of Parkinson’s disease of recombinant GDNF carrying this deletion was reduced compared to the wild-type protein, probably due to its lower retention at the site of administration [46] as previously mentioned. Overall, these studies show some similarity in the role of heparin and HS in BMP and GDNF signalling and bioavailability.

However, a major complication in attempting to assess the role of HS in BMP signalling within the tissues is the presence of the various BMP antagonist proteins, a high proportion of which themselves bind with high affinity to heparin and HS. Investigations in this area overall remain limited, but several instances provide some insight. Thus, as referred to above, the crystal structure of the follistatin isoform 288 complexed with myostatin (GDF-8) reveals a continuous electropositive surface generated at the interface between the two proteins. This accounts for the higher heparin binding affinity of the complex compared to the free follistatin [40,58]. Since there is no observation of the binding of myostatin to heparin/HS, binding to follistatin 288 would appear to be a mechanism for tethering the cytokine to cell surface HS. The antagonist Noggin, by binding to cell surface HS proteoglycans, is thought to reduce the diffusion of BMP-4 [23] thereby establishing morphogenetic gradients in the embryo. Interestingly, a mutation within the heparin binding site of Noggin which reduces its affinity underlies the congenital disorders of proximal subphalagism and conductive hearing loss [59], strongly implicating heparin-binding in the functioning of Noggin during development.

The high resolution crystal structure of gremlin-2 complexed with GDF-5 [60] shows a very different orientation of the two proteins in the complex compared to the noggin-BMP-7 structure (Figure 4). In the former, the gremlin dimers are perpendicular to, and at the tips of the GDF-5 dimers, potentially allowing for the assembly of large repeat alternating “daisy chain” complexes of the two proteins, and formation of such multimers has been demonstrated in vitro [60]. Modelling the gremlin-2/BMP-2 complex on the gremlin-2/GDF-5 structure shows a large continuous basic surface patch at the interface of the antagonist and cytokine which may explain why the complex binds to heparin with higher affinity than either protein partner alone [22]. On the basis of these data, it may be suggested that HS chains in the tissues would promote the assembly of large gremlin-2/cytokine complexes with repeating heparin/HS binding sites would give rise to stable depots of the two proteins in the extracellular matrix and on cell surfaces close to their sites of secretion.

## 7. Heparin/HS Binding in the Therapeutic Applications of TGF-β Superfamily Cytokines

There has been widespread interest in exploiting the cellular regulatory activities of TGF-β cytokines in the therapy of a range of chronic diseases. A prominent instance of this is the clinical use of recombinant BMP-infused cements for non-unionising bone fractures and in the fusing of spinal vertebrae. Several groups are investigating whether the incorporation of heparin or HS into such biomaterials might provide for better bone growth outcomes by improving the bioavailability of BMP-2 through its slow release [61,62,63]. Heparin coatings for the titanium surfaces of skeletal implants are also under active consideration for the same reason [64]. Another area of interest is the potential therapeutic use of GDNF and related neurotrophins as neuroprotective and neuroregenerative agents in nervous system disease or injury. Although administration of recombinant GDNF has proved highly effective in rodent models of Parkinson’s disease, a large clinical trial proved unsuccessful [65]. One major issue here is likely to be inadequate delivery of GDNF throughout the larger structures of human brain. This being the case, neurotrophin binding to HS may be disadvantageous in this application. Indeed, recently a variant of neurturin with reduced affinity for HS was found to diffuse further through brain and to be effective in regenerating dopaminergic nerve fibres a rat model of Parkinson’s disease than GDNF [66]. In conclusion, where a TGF-β family cytokine binds heparin/HS, this property will be an important consideration in any potential therapeutic applications.

## Figures and Tables

**Table 1:** Heparin-binding affinity estimates of TGF-β superfamily cytokines.

Protein	Heparin Affinity		Reference
TGF-β1	HAC	≥0.5 M	[13]
TGF-β2	HAC	≥0.5 M	[13]
BMPs/GDFs			
BMP-2	SPR	*K*_d_ 20 nM	[14]
BMP-4	n.d.		-
BMP-6	n.d.		-
BMP-7	HAC	0.5 M	[15]
BMP-14/GDF-5	SPR	*K*_d_ 50 nM	[16]
BMP-15/GDF-9	HAC	≈1.0 M	[17]
Neurotrophins			
GDNF	HAC	0.8 M	[18]
	SPR	*K*_d_ 23 nM	[19]
Artemin	HAC	23 nM	[18]
	SPR	*K*_d_ 45 nM	[19]
Neurturin	HAC	1.2 M	[18]
	SPR	*K*_d_ 115 nM	[19]
Can family antagonists			
Gremlin-1	HAC	0.8 M	[20]
	SPR	*K*_d_ 20 nM	[21]
Gremlin-2/PRDC	HAC	0.67 M	[22]
Sclerostin	n.d		-
Other antagonists			
Noggin	HAC	0.8 M	[23]
Chordin	HAC	1.0 M	[24]
Follistatin (288 isoform)	SPR	*K*_d_ 1.0 M	[25]

The techniques have been employed: heparin affinity chromatography, HAC, and surface plasmon resonance (SPR). HAC employs a NaCl gradient to elute the bound cytokine, and the concentration of salt required is given. This approach therefore investigates the ionic component of the binding interaction. For SPR, the estimated dissociation constant is presented. In comparing estimates from the different studies, it must be borne in mind that different laboratories will have used a variety of heparin immobilisation procedures and different batches of heparin.
